# First-trimester smoking cessation in pregnancy did not increase the risk of preeclampsia/eclampsia: A Murmansk County Birth Registry study

**DOI:** 10.1371/journal.pone.0179354

**Published:** 2017-08-10

**Authors:** Olga A. Kharkova, Andrej M. Grjibovski, Alexandra Krettek, Evert Nieboer, Jon Ø. Odland

**Affiliations:** 1 Department of Community Medicine, Faculty of Health Sciences, UiT The Arctic University of Norway, Tromsø, Norway; 2 International School of Public Health, Northern State Medical University, Arkhangelsk, Russia; 3 Department of International Public Health, Norwegian Institute of Public Health, Oslo, Norway; 4 Department of Preventive Medicine, International Kazakh-Turkish University, Turkestan, Kazakhstan; 5 Department of Biomedicine and Public Health, School of Health and Education, University of Skövde, Skövde, Sweden; 6 Department of Internal Medicine and Clinical Nutrition, Institute of Medicine, Sahlgrenska Academy at University of Gothenburg, Gothenburg, Sweden; 7 Department of Biochemistry and Biomedical Sciences, Hamilton, ON, Canada; 8 School of Health Systems and Public Health, Faculty of Health Sciences, University of Pretoria, Pretoria, South Africa; Univesity of Iowa, UNITED STATES

## Abstract

**Background:**

Although prior studies have shown that smoking reduces preeclampsia/eclampsia risk, the consequence of giving up this habit during pregnancy should be assessed. The aims of the current study were threefold: (i) describe maternal characteristics of women with preeclampsia/eclampsia; (ii) examine a possible association between the number of cigarettes smoked daily during pregnancy and the development of this affliction; and (iii) determine if first-trimester discontinuation of smoking during pregnancy influences the risk.

**Methods:**

A registry-based study was conducted using data from the Murmansk County Birth Registry (MCBR). It included women without pre-existing hypertension, who delivered a singleton infant during 2006–2011 and had attended the first antenatal visit before 12 week of gestation. We adjusted for potential confounders using logistic regression.

**Results:**

The prevalence of preeclampsia/eclampsia was 8.3% (95%CI: 8.0–8.6). Preeclampsia/eclampsia associated with maternal age, education, marital status, parity, excessive weight gain and body mass index at the first antenatal visit. There was a dose-response relationship between the number of smoked cigarettes per day during pregnancy and the risk of preeclampsia/eclampsia (adjusted OR_1-5 cig/day_ = 0.69 with 95%CI: 0.56–0.87; OR_6-10 cig/day_ = 0.65 with 95%CI: 0.51–0.82; and OR_≥11 cig/day_ = 0.49 with 95%CI: 0.30–0.81). There was no difference in this risk among women who smoked before and during pregnancy and those who did so before but not during pregnancy (adjusted OR = 1.10 with 95%CI: 0.91–1.32).

**Conclusions:**

Preeclampsia/eclampsia was associated with maternal age, education, marital status, parity, excessive weight gain, and body mass index at the first antenatal visit. There was a negative dose-response relationship between the number of smoked cigarettes per day during pregnancy and the odds of preeclampsia/eclampsia. However, women who gave up smoking during the first trimester of gestation had the same risk of preeclampsia/eclampsia as those who smoked while pregnant. Consequently, antenatal clinic specialists are advised to take these various observations into account when counselling women on smoking cessation during pregnancy.

## Introduction

Preeclampsia and eclampsia are common complications of pregnancy that annually affect 8,370,000 women worldwide [1, 2]. Preeclampsia in a woman who before 20 weeks’ gestation was previously normotensive is defined by the presence of hypertension (systolic or diastolic blood pressures of >140mm and >90 mm Hg) in combination with proteinuria with or without oedema [3–5]. Eclampsia constitutes the onset of seizures in a woman with preeclampsia [6].

The crude incidences of preeclampsia between 2002 and 2010 in the Eastern Mediterranean (EMRO) and Western Pacific (WPRO) WHO regions, respectively, were 1.2 to 4.2% [7]. When a logistic model using bootstrapped data was employed to estimate the incidence of preeclampsia using country macroeconomic indicators of health care and population characteristics for adjustment, the overall estimate for all WHO regions was 4.6% (with a 95% uncertainty range of 2.7–8.2); in the EMRO and AFRO WHO regions the incidences were 1.0 and 5.6%, respectively. In the EURO WHO region excluding Russia (due to missing data), the crude incidence of preeclampsia was 3.8%, with a corresponding model-based incidence (in %) of 5.3 (1.8–9.3) [7]. The overall prevalence of preeclampsia/eclampsia in Russia depends on the year of measurement and the federal district [8–10]. For example, during 2005–2010 the prevalence ranged from 2.1 to 1.4% in the Southern and Northern Caucasians, 0.1–0.6% in Far Eastern federal districts and 2.4–2.6% in the Northwestern and Ural districts.

We recently demonstrated a high prevalence of smoking before and during pregnancy in Northwest Russia, with only one of four smokers quitting during pregnancy [11]. This raised concern since tobacco smoking during pregnancy increases the risk of placental complications including placenta previa [12] and placental abruption [13], as well as preterm birth [14] and low birth weight [15, 16]. Maternal smoking also associates with increased perinatal morbidity and mortality, while in the general population maternal smoking constitutes a risk factor for many chronic diseases including cardiovascular disease, diabetes, and inflammatory diseases [17, 18].

Cigarette smoking appears to reduce the risk of preeclampsia and eclampsia [19–21]. However, there is no consistent evidence to determine if giving up smoking during pregnancy influences this protective effect. Some studies suggest a reduced risk [22–24], while others show no effect [21, 25, 26]. This discrepancy could be a consequence of variation in study design, sample selection or its size. Moreover, most studies do not adjust for potential confounders such as socioeconomic status [22, 23] and weight gain during pregnancy [21, 22, 24, 26]. In the present study, we employ registry data to examine potential modifiers of the impact maternal smoking has on the risk of preeclampsia/eclampsia, including putative confounding factors. Our aims are to: (i) describe maternal characteristics of women with preeclampsia/eclampsia; (ii) examine a potential association between the number of smoked cigarettes per day during pregnancy and the development of preeclampsia/eclampsia; and (iii), determine if first-trimester smoking cessation during pregnancy influences the risk of preeclampsia/eclampsia.

## Materials methods

### Study setting, design and sample size

The study was conducted in Murmansk County, which is a federal subject of the north-western part of Russia. The County borders with the Republic of Karelia in Russia, Lapland Region in Finland and Finnmark County in Norway. Murmansk County is surrounded in part by the Barents Sea and White Sea.

**Fig 1:**
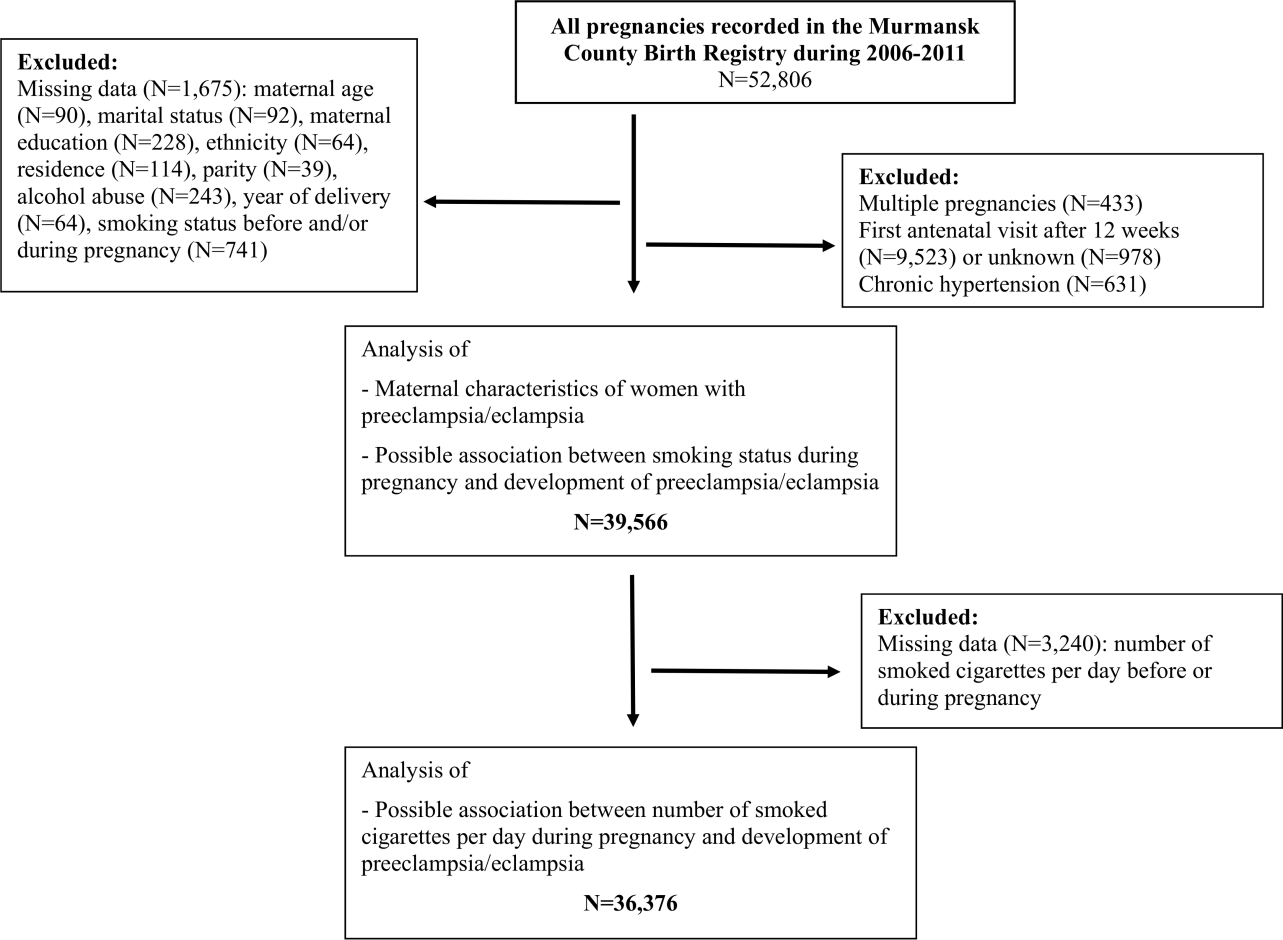
Sampling and analysis details.

The study population consisted of all women who were registered in the Murmansk County Birth Registry (MCBR). Its background details are described elsewhere [11], including implementation and quality control details [27]. For the purposes of this study we excluded women if they had a multiple pregnancy, pre-existing hypertension, or had their first antenatal visit after week 12 of gestation. Three specific issues are the focus of the current publication: (i) maternal characteristics of the study participants (N = 39 566); (ii) the association between the numbers of smoked cigarettes per day and incidence of preeclampsia/eclampsia (N = 36 376); and (iii) and the impact on the latter of the first-trimester smoking cessation during pregnancy (N = 39 566). Sampling and analysis details are provided in Fig 1.

### Data collection

Based on medical records and personal interviews with the expectant mothers, the MCBR contained information on maternal characteristics including age, ethnicity, residence, educational level, marital status, parity, alcohol abuse as diagnosed by a doctor, self-reported smoking before and during pregnancy, and maternal weight and height measured at the first antenatal visit. Information in the MCBR on preeclampsia/eclampsia occurrence, excessive weight gain during pregnancy and year of delivery were derived from individual obstetric journals.

Preeclampsia and eclampsia were classified according to the International Classification of Diseases, tenth revision (ICD-10) [28]. Preeclampsia (ICD-10 codes O14.0 “mild to moderate preeclampsia”; O14.1 “severe preeclampsia”) is a pregnancy-induced hypertensive state that occurs after 20 weeks of gestation. It is characterized by hypertension (blood pressure of 140/90 or higher), along with oedema and proteinuria (300 mg of protein in a 24-hour urine sample) [8, 29]. Eclampsia (ICD-10 code O15.0) involves convulsions and coma in pregnant or puerperal women along with hypertension, oedema, and proteinuria.

We analysed preeclampsia (N = 3276) and eclampsia (N = 5) cases together because of the limited number of cases of eclampsia. The variable “preeclamsia/eclampsia” (N = 3281) was treated as binary variable.

In terms of smoking status during pregnancy, women were grouped as smokers (did so before and during pregnancy), quitters (smoked before but not during pregnancy), or non-smokers (did not smoke before nor during pregnancy). Smoking status was assessed during the first antenatal visit. Number of smoked cigarettes per day during pregnancy was taken as a categorical variable, specifically as 0, 1–5, 6–10, and ≥11.

Maternal age was also treated as a categorical variable, namely: ≤19 years, 20–24 years, 25–29 years, 30–34 years and ≥35 years. Place of residence was registered as urban or rural. Ethnicity was dichotomized into Russian and other. Maternal education was divided into three categories: *as less than university* that included primary (0–9 years of schooling), secondary (10–11 years of schooling) and vocational training, *university* and *unknown*. Marital status was classified as married, cohabitating and single, with the latter including divorced and widows. Parity was categorized as 0, 1, and ≥2 previous deliveries. Alcohol abuse was recorded as either yes or no. Year of delivery was denoted by the exact calendar year.

Body mass index (BMI) was calculated for the women’s weight at the first antenatal visit (kg) divided by height (m^2^). By BMI, women were classified into five groups: underweight (≤18.4 kg/m^2^), normal weight (18.5–24.9 kg/m^2^), overweight (25.0–29.9 kg/m^2^), obese (≥30.0 kg/m^2^), and unknown.

Excessive weigh gain was defined as weight gain during pregnancy of > 18 kg in underweight women, > 16 kg in normal weight women, > 11.5 kg in overweight women, and ≥ 6 kg in obese women. Excessive weight gain in pregnancy (ICD-10 code O26.0) was dichotomized as yes and no.

### Data analysis

Pearson’s chi-squared tests were used to analyse the categorical variables for differences. By logistic regression we examined the effects of giving up smoking during pregnancy on the risk of preeclampsia/eclampsia, and any association between preeclampsia/eclampsia and daily numbers of smoked cigarettes while pregnant. Both crude and adjusted odds ratios (ORs) and their 95% confidence intervals (CI) are reported. All statistical analyses were conducted using SPSS, version 22 (SPSS Inc., Chicago, IL).

### Ethical considerations

The study was approved by the Ethical Committee of Northern State Medical University, Arkhangelsk, Russia, and the Norwegian Regional Committee for Medical and Health Research Ethics (REC-North), Tromsø, Norway.

## Results

### Maternal characteristics of women with preeclampsia/eclampsia

Of the 39 566 participants in our study, 8.3% (95% CI: 8.0–8.6) had preeclampsia/eclampsia during their current pregnancy. Occurrence was more prevalent in women ≥35 years old, having less than university education, and who were nulliparous and single (Table 1). Preeclampsia/eclampsia developed more frequently among women who were obese at the first antenatal visit and those with normal weight. Furthermore, risk of preeclampsia/eclampsia was 6.7% among women who smoked in pregnancy and 8.7% among those who did not (p<0.001). The proportion of women with preeclampsia/eclampsia decreased with the number of cigarettes smoked per day (p<0.001), while ethnicity, residence (urban *versus* rural) and alcohol abuse had no impact.

**Table 1:** Maternal characteristics of the study participants (N = 39 566).

Maternal characteristics	Total number	Women without preeclampsia/eclampsia		Women with preeclampsia/eclampsia		P-level
		N	%	N	%	
*Maternal age (years)*						0.001
≤ 19	2112	1943	92.0	169	8.0	
20–24	11 532	10 601	91.9	931	8.1	
25–29	13 801	12 723	92.2	1078	7.8	
30–34	8724	7955	91.2	769	8.8	
≥ 35	3397	3063	90.2	334	9.8	
*Residence*						0.223
Urban	34 349	31 478	91.6	2871	8.4	
Rural	5217	4807	92.1	410	7.9	
*Ethnicity*						0.329
Russian	38 043	34 878	91.7	3165	8.3	
Other	1523	1407	92.4	116	7.6	
*Education*						<0.001
Less than university	24 897	22 797	91.6	2100	8.4	
University	14 488	13 340	92.1	1148	7.9	
Unknown	181	148	81.8	33	18.2	
*Marital status*						<0.001
Married	30 402	27872	91.7	2530	8.3	
Cohabitation	6107	5705	93.4	402	6.6	
Single	3057	2708	88.6	349	11.4	
*Parity*						<0.001
0	22 489	20 481	91.1	2008	8.9	
1	14 742	13 649	92.6	1093	7.4	
≥2	2335	2155	92.3	180	7.7	
*Alcohol abuse*						0.844
No	39 499	36 224	91.7	3275	8.3	
Yes	67	61	91.0	6	9.0	
*BMI*						<0.001
Underweight	2478	2404	97.0	74	3.0	
Normal weight	25 836	24 165	93.5	1671	6.5	
Overweight	7331	6432	87.7	899	12.3	
Obese	2918	2374	81.4	544	18.6	
Unknown	1003	910	90.7	93	9.3	
*Excessive weight gain*						<0.001
No	37 148	34001	91.5	3147	8.5	
Yes	2418	2284	94.5	134	5.5	
*Smoking status during pregnancy*						<0.001
Non-smoker	30 690	28 019	91.3	2671	8.7	
Quitter	2534	2350	92.7	184	7.3	
Smoker	6342	5916	93.3	426	6.7	
Number of smoked cigarettes per day during pregnancy*						<0.001
0	33 219	30 364	91.4	2855	8.6	
1–5	1411	1321	93.6	90	6.4	
6–10	1333	1253	94.0	80	6.0	
≥ 11	413	396	95.9	17	4.1	

* Total number is 36 376 because of missing data

Calculated using chi-squared test

### Association between daily numbers of smoked cigarettes during pregnancy and development of preeclampsia/eclampsia

Non-smokers both before and during pregnancy had a greater risk of preeclampsia/eclampsia compared to smokers (Table 2). A dose-response relationship was evident between the number of cigarettes smoked per day during pregnancy and the risk of preeclampsia/eclampsia (p_trend_<0.001). Note that pregnant women who smoked 1–5, 6–10 or ≥11 cigarettes per day during pregnancy had decreased odds of having preeclampsia/eclampsia compared to non-smokers (crude OR_1-5cig._ of 0.72 with 95% CI: 0.58–0.90; for OR_6-10cig._ of 0.68 with 95% CI: 0.54–0.85; and for OR_≥ 11cig._ of 0.46 with 95% CI: 0.28–0.74, respectively). Adjustment for potential confounders did not change the association.

**Table 2:** Association between daily numbers of smoked cigarettes during pregnancy and preeclampsia/eclampsia among women with singleton pregnancies in Murmansk County, Northwest Russia (N = 36 376).

Variable	Crude			Adjusted^1^		
	OR	95% CI	p_trend_	OR	95% CI	p_trend_
*Number of smoked cigarettes per day during pregnancy*			<0.001			<0.001
0	1.00			1.00		
1–5	0.72	0.58–0.90		0.69	0.56–0.87	
6–10	0.68	0.54–0.85		0.65	0.51–0.82	
≥ 11	0.46	0.28–0.74		0.49	0.30–0.81	

^1^ OR adjusted for variables maternal age, residence, ethnicity, marital status, parity, alcohol abuse, year of delivery, body mass index and excessive weigh gain

### Quitting smoking during pregnancy and risk of preeclampsia/eclampsia

Women who smoked before but not during pregnancy had lower risk of having preeclampsia/eclampsia compared to those who did not smoke before and during pregnancy [adjusted OR of 0.80 (95% CI: 0.68–0.94)]. However, there was no significant difference in the occurrence of preeclampsia/eclampsia among women smoking before and during pregnancy and those who smoked before, but not during pregnancy―either before or after adjustment for other maternal characteristics (Table 3).

**Table 3:** Association between smoking status during pregnancy and preeclampsia/eclampsia among women with singleton pregnancies in Murmansk County, Northwest Russia (N = 39 566).

Variable	Crude		Adjusted^1^	
	OR	95% CI	OR	95% CI
Smoking before and during pregnancy	1.00		1.00	
Smoked before, but not during pregnancy	1.09	0.91–1.30	1.10	0.91–1.32
Did not smoke both before and during pregnancy	1.32	1.19–1.47	1.37	1.23–1.54

^1^ OR adjusted for variables maternal age, residence, ethnicity, marital status, parity, alcohol abuse, year of delivery, body mass index and excessive weigh gain

## Discussion

### Main findings

Preeclampsia/eclampsia was more common in older, single, less educated and primiparae women and those who were overweight or obese at the first antenatal visit and had no excessive weight gain during pregnancy. Non-smokers both before and during pregnancy had a greater risk of preeclampsia/eclampsia compared to smokers. A dose-response relationship was evident between the number of cigarettes smoked per day during pregnancy and the risk of preeclampsia/eclampsia. Furthermore, discontinuance of smoking during pregnancy did not increase the risk of preeclampsia/eclampsia.

### Data interpretation and comparisons with previous studies

The observed preeclampsia/eclampsia prevalence in Murmansk County of 8.3% is higher than previous estimates [7–10, 23, 30]. This could reflect different definitions and differing proportions of primiparae [31]. Preeclampsia is more common in primiparae than in multiparae, which is a potential reason for discrepancies in parity among countries. Furthermore, regional data are often different from national figures, as they reflect the variation of preeclampsia/eclampsia within one country. For example in St. Petersburg (located in the Northwest federal district of Russia), the prevalence of preeclampsia/eclampsia in 2005 was 7.1%, while it was 8.6% in Orenburg County (Volga federal district), 10.5% in Kurgan County (Ural federal district) and <0.1% in Vladimir County (Central federal district) and Vologda (Northwest federal district) [10].

Our observations that the risk of preeclampsia/eclampsia increased in women who are ≥35 years old, have less education than university, are single, primiparae and overweight or obese at the first antenatal visit are consistent with earlier studies [21, 31, 32].

Our finding of a 2-fold protective effect for the development of preeclampsia/eclampsia in women who smoked more than ≥ 11 cigarettes per day relative to non-smokers supports previous reports [19–21]. According to Linqvist et al [32], moderate smokers (1–9 cigarettes per day) are characterized by a lower incidence of preeclampsia compared to non-smokers. Similarly, Yang et al [33] report an inverse exposure-response association as does Bainbridge et al [34]. Venditti et al [35] state that the use of carbon monoxide (CO) could prevent the development of hypertension and proteinuria in a rodent model of preeclampsia. Bainbridge et al [34] suggest that CO, a combustible product in cigarettes, may be the active agent. More recently Zhai et al [36] demonstrated an inverse correlation between increased environmental ambient CO and preeclampsia. However, any interpretations must consider that the pathogenesis of preeclampsia is complex as it appears to involve genetic, immunological and environmental factors [37].

Tobacco smoking during pregnancy can potentially impact angiogenic factors, endothelial function and the immune system, which could lead to a lower risk of preeclampsia. However, this protective role is most likely explained by CO’s biological role in heme-degradation processes including the promotion of anti-inflammatory and pro-angiogenic effects [38–40]. On the other hand, the mechanisms underlying the increased risk of preeclampsia among previous and passive smokers remain unclear. Luo et al [41] suggest that this could be due to adverse chronic effects of low tobacco exposure in the absence of significant exposure to a transient protective factor such as CO in association with current smoking.

Our data suggest that discontinuing smoking after pregnancy awareness did not alter the risk of preeclampsia/eclampsia statistically speaking. By contrast, some studies demonstrate a lower incidence of preeclampsia among women who stop smoking at the beginning of pregnancy compared to those who never smoked [22, 23]. Neither do our findings align with those of England et al [26] in their randomized clinical trial “Calcium for Preeclampsia Prevention” (N = 4,589). They observed that the incidence of preeclampsia among women who stopped smoking 13–21 weeks before pregnancy was similar to that among women who never smoked. This difference is likely related to whether cessation of smoking occurred after pregnancy recognition rather than well before pregnancy.

Studies based on the measurements of biomarkers of smoking such as plasma or salivary cotinine demonstrate diverse findings as well [41, 42]. A prospective pregnancy cohort study defined smoking status according to plasma cotinine, and found that previous and passive smokers compared to non-smokers were almost six-fold more likely to exhibit preeclampsia [41]. However, women who smoked during their current pregnancy had almost the same risk of preeclampsia as non-smokers. Another study did not show significant differences in preeclampsia rates using lower cutoffs of cotinine exposure [42].

Mainstream smoke contains multiple toxic chemicals in addition to nicotine and CO that are volatile, e.g., acetaldehyde, hydrogen cyanide, nitric acid, acetone, ammonia, hydrogen sulfide, hydrocarbons, nitrosamines, and carbonyl compound [17]. The smoke particulate phase also contains multiple toxicants such as carboxylic acids, phenols, terpenoids, paraffin waxes, tobacco-specific nitrosamines, polyaromatic hydrocarbons. Clearly smoking during pregnancy is not recommended in the context of reported detrimental concerns that include increases in perinatal mortality, abruptio placenta, intrauterine growth retardation [43, 44] and birth defects (e.g., oral clefts) [45].

### Limitations and strengths

All data regarding smoking status was self-reported, which may have contributed to misclassification, and thus would constitute measurement bias. If exposure misclassification did occur in our study it most likely was among smoking women who falsely reported that they stopped after pregnancy recognition, or among those who gave up smoking in the first trimester during pregnancy but subsequently resumed this practise. This type of misclassification would have decreased the risk of preeclampsia/eclampsia among those who gave up smoking while pregnant. However, we found that women who reported that they gave up smoking after pregnancy recognition had the same risk of preeclampsia/eclampsia as women who indicated they smoked before and during pregnancy. Our information about smoking behaviour was collected during the first antenatal visit. Räisänen et al [46] consider that gathering smoking status information during the first antenatal visit is more reliable than assessing it at the time of birth. Although we did not have data about the duration of tobacco smoke exposure, we did control for the number of cigarettes smoked daily during pregnancy.

The major strength of our study is that the data represent almost the total population of pregnant women in Murmansk County who delivered a singleton infant during 2006–2011 and had the first antenatal visit before 12 weeks of gestation. The registry data were collected in clinics and the number of births registered in the MCBR comprised 98.8% of the official number of births recorded by the Health Department in Murmansk County [27]. This allows generalizing of the results at the population level. Furthermore, quality controls in 2006–2007 suggested that the validity of data in MCBR is sufficient for epidemiological studies [27]. In contrast to previous studies [19–21], we had the ability to control for the influence of possible confounding factors such as social-demographic characteristics of pregnant women, body mass index at the first antenatal visit and excessive weight gain in pregnancy.

Our findings and perspective may provide clinicians with a better understanding of the necessity of promoting women to discontinue smoking during pregnancy, as well as rationale for the benefits of doing so early in the pregnancy. Clinicians might communicate that the risk of preeclampsia/eclampsia will not be affected by giving up smoking during pregnancy.

## Conclusions

In summary, we found that preeclampsia/eclampsia was associated with some maternal characteristics, such as maternal age, education, marital status, parity, excessive weight gain, and body mass index at the first antenatal visit. Our study demonstrates that maternal smoking was inversely associated with preeclampsia/eclampsia. Moreover, increased number of daily smoked cigarettes during pregnancy decreased odds of preeclampsia/eclampsia. Interesting, if women quit smoking during pregnancy they had the same risk of preeclampsia/eclampsia as those who smoked while pregnant. Even though our findings imply that giving up smoking does not alter the reduced risk of preeclampsia/eclampsia, it would most likely mitigate known smoking-related risks to the mother and the unborn. This advice might be shared during the first antenatal visit.

## Abbreviations

MCBR: Murmansk County Birth Registry
WHO: World health organization
EMRO: Eastern Mediterranean region
AFRO: African region
EURO: European region
ICD: International classification of diseases
BMI: Body mass index
CO: carbon monoxide
